# A-C/Au Film with Low Humidity Sensitivity of Friction by Forming Au Transfer Film

**DOI:** 10.3390/ma17204941

**Published:** 2024-10-10

**Authors:** Lulu Pei, Li Ji, Hongxuan Li, Haichao Cai, Yujun Xue

**Affiliations:** 1 School of Mechatronics Engineering, Henan University of Science and Technology, Luoyang 471003, China; 2 Henan Key Laboratory for Machinery Design and Transmission System, Henan University of Science and Technology, Luoyang 471003, China; 3 Collaborative Innovation Center of Henan Province for High-End Bearing, Henan University of Science and Technology, Luoyang 471000, China; 4 State Key Laboratory of Solid Lubrication, Lanzhou Institute of Chemical Physics, Chinese Academy of Sciences, Lanzhou 730000, China

**Keywords:** carbon materials, wear and tribology, Au transfer film, humidity sensitivity

## Abstract

Amorphous carbon is recognized as an excellent lubricating material; however, its tribological properties are significantly influenced by humidity. To elucidate the mechanism underlying this humidity dependence and to propose a novel enhancement method, we investigated and compared the tribological properties of hydrogenated amorphous carbon (a-C:H) and amorphous carbon/gold (a-C/Au) composite films. First, the friction coefficient of these carbon films under different humidity conditions was tested using a rotational ball-on-disk tribometer. Subsequently, we analyzed the morphology and structure of the sliding interface employing optical microscopy (OM), Raman spectroscopy, transmission electron microscopy (TEM), and high-resolution transmission electron microscopy (HRTEM). Finally, first-principle calculations were carried out to calculate the adsorption energy of water molecules on different surfaces. The results indicate that the friction coefficient of a-C:H film and the area of transfer film increase with the increase of humidity. This phenomenon can be attributed to the fact that water molecules enhance the interaction between the a-C:H film and steel counterfaces. Notably, in contrast, the friction coefficient of a-C/Au film demonstrates low sensitivity to humidity due to the formation of an Au transfer film that exhibits weak interaction with water molecules. These findings provide a promising strategy for developing environment-adaptive amorphous carbon films and play an important role in promoting the development of intelligent lubricating film.

## 1. Introduction

Amorphous carbon is well-known for its excellent mechanical and tribological properties, making it a popular lubricating material [1,2]. Specifically, hydrogenated amorphous carbon (a-C:H) exhibits superlubricity in vacuum or inert environments [3]. However, its tribological properties are highly sensitive to humidity [4,5], which has seriously affected its multi-environment application.

Many experiments have demonstrated that the humidity dependence of a-C:H films can be attributed to three factors [6,7,8]. First, adsorbed water induces adhesion and capillary forces. Second, it hinders the formation of a carbon transfer film. Lastly, easily-sheared carbon-hydrogen layers are oxidized into difficult-to-shear carbon-oxygen layers. Thus, inhibiting the oxidation of carbon or forming a frictional interface with lower shear force by doping other elements are mainstream strategies for enhancing the film’s resistance to humidity [9,10,11,12,13]. Wang et al. [9] improved the humidity dependence of a-C:H films by spin-coating an h-BN layer on surface of a-C film, showing that the underlying mechanism attributed to h-BN can enhance the graphitization process to form more graphite-like carbon. The friction coefficient of a-C:H films under high humidity decreases by doping Si; this is because it can react with water molecules to form a silica-like tribolayer with a lower shear force [11]. Although these studies have improved the tribological properties of amorphous carbon film under humidity environments, its friction mechanism under high humidity is still controversial, and there is a lack of a universal method to improve the humidity sensitivity of amorphous carbon films.

In our previous study, we found the interaction between carbon film and counterface is key to its tribological properties [14]. Thus, weakening the interaction between water molecules and the sliding interface is also an effective method to decrease the friction coefficient under high humidity. Meanwhile, we discovered that the Au of amorphous carbon/Au (a-C/Au) composite film can migrate to the steel counterface during friction [15]. Au is an inert metal with a weak interaction with water molecules, which has great potential to maintain a stable sliding interface regardless of humidity. Inspired by this result, this study compared the friction coefficient of a-C:H and a-C/Au films under different humidity environments, analyzed the structural evolution of the friction interface, and revealed the lubricating mechanisms of amorphous carbon films under different humidity.

## 2. Materials and Methods

The a-C:H and a-C/Au films were deposited on polished SUS-304 steel substrates by an unbalanced magnetron sputtering system. Before depositing the amorphous carbon film, a Ti layer (270 nm), which has a strong adhesion force with steel substrates, was deposited. Then, the a-C/Au layer was deposited by sputtering graphite and Au targets and a-C:H layer was deposited by sputtering graphite targets. The deposition parameters of the amorphous carbon layer and film properties are shown in Table 1, and the more detailed deposition process has been previously described [15]. Friction and wear tests were conducted using a rotational ball-on-disk tribometer (CSM) at different relative humidities (RH; 35%, 60% and 75%), and the ambient temperature was maintained at 25 ± 1 °C for all tests. The tribometer is equipped with a chamber, and humidity is controlled by the number of beakers filled with water in the chamber. The friction test begins when the hygrometer remains stable after twenty minutes. The counterface is a GCr15 steel ball with a diameter of 6 mm. Test parameters included a load of 5 N, a sliding radius of 5 mm, and a rotational speed of 300 rpm. Each friction test was stopped when reaching 10,000 sliding cycles and repeated at least three times.

The Au content of a-C/Au composite film was tested by a multifunctional X-ray photoelectron spectrometer with Al-Kα as the excitation source (XPS, Perkin-Elmer PHI-5702, Waltham, MA, USA). The surface roughness and thickness of films were analyzed by a 3D non-contact surface profilometer (Micro XAM, KLA, Milpitas, CA, USA). The hardness and elasticity modulus of films were measured with a nanoindenter (NHT^2^, CSM, Basel, Switzerland). The wear morphology on the steel ball was observed by an optical microscope (OM, STM6, Olympus, Tokyo, Japan). Raman spectroscopy is an efficient tool to character the amorphous carbon, and it has an obvious asymmetry peak around 1500 cm^−1^. It can be deconvoluted into two peaks identified as D and G centered at around 1380 cm^−1^ and 1560 cm^−1^, respectively. Thus, the structure of transfer film on the surface of the steel counterface was characterized by Raman spectroscopy at a wavelength of 532 nm (Raman, Renishaw, Gloucestershire, UK). Cross-sectional specimens were cut from a steel ball using a focused ion beam (FIB, scios, FEI, Hillsboro, OR, USA), and the specimens obtained were analyzed using a transmission electron microscope (TEM, Talos-F200S, FEI, Hillsboro, OR, USA) and high-resolution transmission electron microscopy (HRTEM, FEI, Hillsboro, OR, USA). The morphology and structure of the wear debris were analyzed by TEM and HRTEM.

The Vienna Ab Initio Package (VASP 6.3.2) [16,17] was employed to perform all spin-polarized density functional theory (DFT) calculations within the generalized gradient approximation (GGA) using the Perdew–Burke–Ernzerhof (PBE) [18] formulation. The projected augmented wave (PAW) potentials [19,20] were chosen to describe the ionic cores and to take valence electrons into account using a plane wave basis set with a kinetic energy cutoff of 450 eV. Using Gaussian smearing and a width of 0.05 eV allows partial occupation of the Kohn Sham orbit. When the energy change is less than 10^−5^ eV, it is considered that the electron energy is self-consistent. A geometry optimization was considered convergent when the energy change was smaller than 0.02 eV Å^−1^. The vacuum spacing in a direction perpendicular to the plane of the structure is 18 Å. The weak interaction was described by the DFT + D3 method using empirical correction in Grimme’s scheme [21,22].

The adsorption energy (E_ads_) was calculated using Equation (1):

E_ads_ = E_total_ − E_substrate_ − E_adsorbate_ (1)

The E_total_, E_substrate_, and E_adsorbate_ represent the energy of adsorption structure, surface (Au(111)/Fe(111)/Graphene/Graphene with defect), and adsorbate (H_2_O), respectively.

## 3. Results

**Figure 1:**
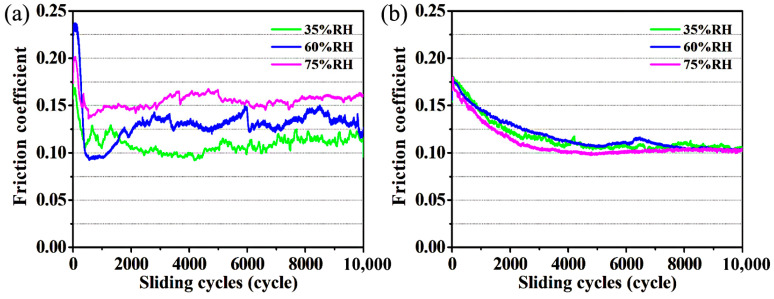
The friction coefficient curves of a-C:H (**a**) and a-C/Au (**b**) films under different humidities.

The friction coefficient curves of a-C:H and a-C/Au films under different humidities are shown in Figure 1. For a-C:H films, the friction coefficient is about 0.1 at 35% RH (Figure 1a). This friction coefficient rises to 0.13 when the RH is increased to 60%, and further increases to 0.15 as the RH continues to rise. These results indicate that the friction coefficient of a-C:H films increases with increasing RH, which is similar to previous studies [23]. Even if the friction coefficient at the later stage of friction stabilizes within a stable range, it fluctuates during the friction process. In addition, the friction coefficient curves at different humidities experienced different running-in processes. We have conducted multiple repeated experiments, and although the initial fluctuations were different, the final friction coefficient was similar under the same conditions. We suspect that it is related to the time of humidity stabilization (it affects the adsorption of water molecules on the surface). These results all proved the humidity sensitivity of the tribological properties of a-C:H film. Differently, the friction coefficient of a-C/Au films does not show a significant change with RH and finally remains stable at around 0.10, but its friction coefficient has a long running-in time (Figure 1b).

**Figure 2:**
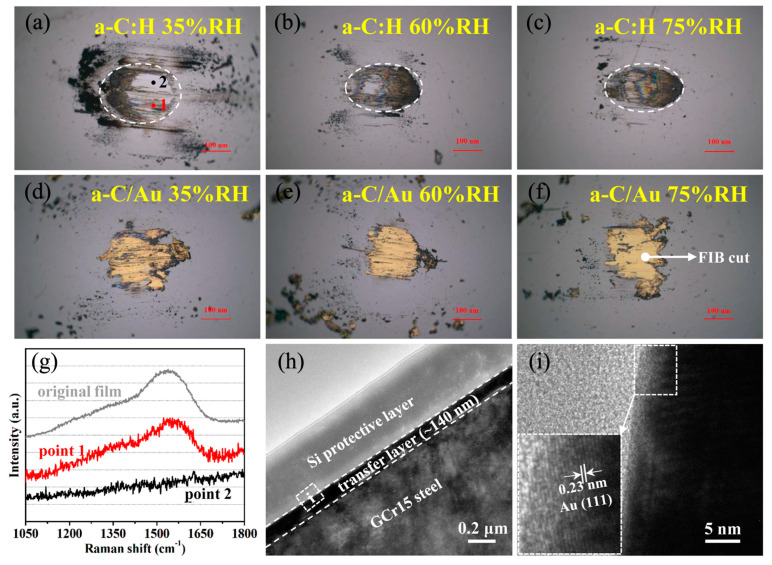
(**a**–**c**) The morphology of wear scar (mark it in dash circles) when a-C:H films slide against steel counterface at different humidities, (**d**–**f**) the morphology of wear scar when a-C/Au films slide against steel counterface at different humidities, (**g**) the Raman spectrum of the position in (**a**), (**h**) the TEM image of the position in (**f**), (**i**) the HRTEM image of the position in (**h**).

The morphology of the wear scar on the steel ball was observed using an optical microscope, and the structure of the transfer film was analyzed using Raman spectroscopy. The wear scar of a-C:H film at 35% RH exhibits two distinct areas (Figure 2a), one of which is similar to the steel substrate (point 2), and there is no obvious carbon signal at this position (Figure 2g). The other area has a color transfer film (point 1), which is a typical amorphous carbon structure. With the RH increase, the area of color transfer film increases (Figure 2b,c), indicating that carbon is easier to transfer to the surface of the steel counterface. However, the formation of the transfer film does not correspond to a low friction coefficient, which is inconsistent with the transfer film theory [24]. The morphology of the transfer film of a-C/Au film is completely different from that of a-C:H film. A yellow transfer layer that is relatively uniform and has no significant effect by humidity was observed (Figure 2d–f), and its thickness is about 200 nm (Figure 2h). The yellow transfer film is a dense Au layer, which can be seen through the interplanar spacing, of 0.23 nm (Figure 2i), corresponding to Au(111).

**Figure 3:**
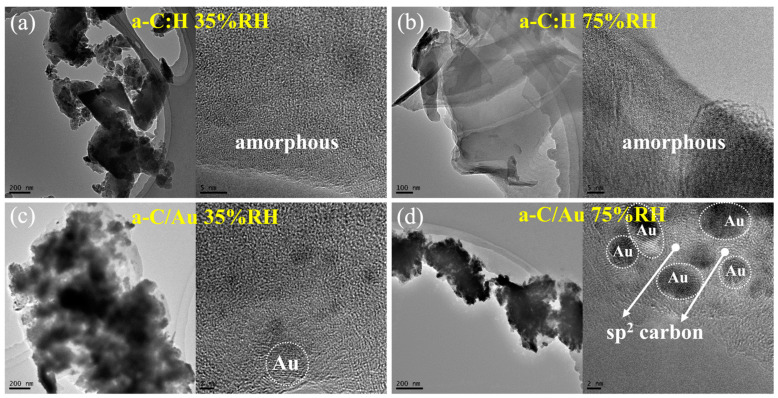
TEM and HRTEM images of wear debris (**a**) when a-C:H film slides against the steel counterface at low humidity, (**b**) when a-C:H film slides against the steel counterface at high humidity, (**c**) when a-C/Au film slides against the steel counterface at low humidity, and (**d**) when a-C/Au film slides against the steel counterface at high humidity.

The morphology and structure of the wear debris were analyzed and are illustrated in Figure 3. For the a-C:H film at low humidity, its wear debris is powdery and exhibits an amorphous structure (Figure 3a). However, the wear debris had flaky morphology at high humidity, similar to that of the original film [25], and also showed an amorphous structure (Figure 3b). The wear debris shows a powdery morphology when a-C/Au film slides against the steel counterface, and it shows an ordered sp^2^ carbon structure (Figure 3c,d), which has more graphite-like carbon than that of the original film [15]. Moreover, the morphology and structure of wear debris have no obvious change with humidity. Many studies have shown that wear debris has a key influence on tribological properties. Gong et al. found that sp^2^ hybridized (graphite-like) carbon is more conducive to reducing the friction coefficient [26]. In addition, our previous study shows that the formation of graphite-like carbon can effectively avoid the effect of unsaturated bonds, thus maintaining a stable friction coefficient [14].

## 4. Discussion

**Figure 4:**
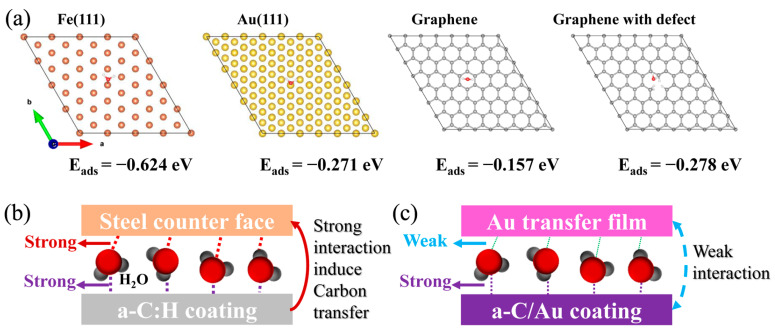
(**a**) The adsorption energy of water molecules on different surfaces, (**b**,**c**) schematic diagram of the interaction of sliding interface in the presence of water molecules.

Water has a significant effect on the interaction between sliding interfaces, consequently influencing its tribological properties. Thus, the adsorption energy of water molecules on the surfaces of Fe (the main component of steel), Au, graphene, and defective graphene (a carbon material) was calculated by means of first principles, as illustrated in Figure 4a. The adsorption energy of water molecules on Fe is the highest (0.624 eV), followed by graphene with a vacancy defect (0.278 eV), the Au (0.271 eV), and the lowest adsorption energy on the perfect graphene (0.157 eV). The results show that defects facilitate the adsorption of water molecules on the carbon materials. Amorphous carbon has a large number of edges and unsaturated bonds, so the adsorption energy of water on its surface is much higher than that of defective graphene.

The high adsorption energy indicates a strong interaction between water molecules and the material. It can therefore be concluded that the water molecules have a strong interaction with the steel and amorphous carbon. The existence of water molecules caused strong interaction between a-C:H film and steel counterface (Figure 4b), and the interaction between sliding interface increases with increasing number of water molecules (rising humidity). Thus, carbon is quickly torn off under a strong interaction, flaky wear debris is formed, and its structural evolution is inhibited, leading the area of transfer film and friction coefficient to increase. For the a-C/Au film, the Au transfer film is formed under friction, and it takes some time for Au to migrate from the film to the counterface surface, so its friction coefficient has a long running-in time [15]. The Au has a weak interaction with water molecules (Figure 4a,c), and the interaction between Au and a-C/Au film has no obvious change with humidity, thus maintaining a stable friction coefficient. In addition, the catalytic action of Au on amorphous carbon facilitates its graphitization transformation, which is more conducive to improving its tribological performance [14]. The phenomenon of Au migration is key to improving the humidity dependence of a-C/Au film, which also provides a new idea for designing environmentally adaptive carbon films.

## 5. Conclusions

This study compared the tribological properties of a-C:H and a-C/Au films at different humidities. The results show the friction coefficient of a-C:H film increases with increasing humidity because of the strong interaction between water molecules and steel and amorphous carbon. Notably, the a-C/Au film shows excellent humidity insensitivity, and its friction coefficient has no obvious change with increasing humidity. The phenomenon can be attributed to the weak interaction between the Au transfer film and water molecules, which is weak enough even at high humidity, thus achieving a stable friction coefficient. Therefore, the formation of Au transfer film provides a new method for improving the humidity sensitivity of the tribological properties of amorphous carbon and provides a new insight for designing intelligent lubricating films.

## Figures and Tables

**Table 1:** The detail deposition parameters and other information of the film.

Film Type	Deposition Pressure (Pa)	Source Gas	The Atomic Content of Au in the Film (at.%)	Film Thickness (μm)	Hardness (GPa)	Elasticity Modulus (GPa)	Surface Roughness (nm)
a-C:H	0.64	Ar:CH_4_ = 45:65	0	1.8	10.00	79.51	4.80
a-C/Au	0.5	Only Ar	16	1.18	5.55	96.84	6.21

## Data Availability

Data will be made available on request.
